# Wilson’s Herald of Health and Farm and Household Help

**Published:** 1874-02

**Authors:** 


WILSON'S HERALD OF HEALTH AND FARM AND HOUSEHOLD HELP.—The December number of this excellent monthly is on our table. It is, as usual, well filled with good advice and instructive articles. Edited by Jno. Stainback Wilson, and published by the Southern Publishing Company, Atlanta, Ga. Price $2.00 a year.

